# Determinants of dental caries among adolescent patients attending Hospitals in West Wollega Zone, Western Ethiopia: A case-control study

**DOI:** 10.1371/journal.pone.0260427

**Published:** 2021-12-02

**Authors:** Tsega Tola Guracho, Emiru Merdassa Atomssa, Obsa Amante Megersa, Tadesse Tolossa

**Affiliations:** 1 World Vision Ethiopia, Nedjo, Oromia, Ethiopia; 2 Department of Public Health, Institute of Health Sciences, Wollega University, Nekemte, Ethiopia; Stomatological Hospital, Southern Medical University, CHINA

## Abstract

**Background:**

Dental caries is a prevalent disease in both developed and developing countries and is a public health problem among adolescents. This study aimed to assess the determinants of dental caries among adolescent patients in the west Wollega Zone, Western Ethiopia.

**Methods:**

A hospital-based unmatched case-control study design was conducted in West Wollega Zone, West Ethiopia. A total of 133 cases and 266 controls participated in this study. Data were collected using pre-tested questionnaires from three hospitals. Epi-info version 7 was used for data entry and was analyzed using SPSS version 20. Frequency, proportion, mean and standard deviation were computed to summarize the data. Statistics are presented using tables and bar graphs. Multivariable binary logistic regression analysis was used to identify the determinants of dental caries at P < 0.05. The adjusted odds ratio with 95% confidence interval (CI) was used to show the strength of association between the predictors and dental caries.

**Results:**

A total of 399 adolescents were enrolled in this study, yielding a response rate of 100%. The study found that, daily consumption of sugared coffee (AOR = 2.91, 95% CI:1.62, 5.23), khat chewing (AOR = 2.90, 95%CI: 1.46, 3.15), daily consumption of bread (AOR = 2.65, 95%CI: 1.44, 4.89), daily consumption of sweet foods (AOR = 2.04, 95%CI:1.19, 3.48), living in urban areas (AOR = 1.86, 95%CI:1.09, 3.15), and daily tooth brushing using toothpaste or stick (AOR = 0.48;95%CI, 0.28, 0.81) were significantly associated with dental caries among adolescents.

**Conclusions and recommendations:**

In this study, drinking sugared coffee, daily consumption of bread, khat chewing, sugary food consumption, living in urban areas, and daily tooth brushing using toothpaste were significantly associated with dental caries. Therefore, improving dietary and drinking habits and strengthening regular tooth brushing are important for reducing dental caries among adolescents.

## Background

Oral diseases are the major public health problem for many countries affecting individuals throughout their lifetime and are common risk factors for non-communicable diseases [1]. Dental caries is the world’s most prevalent, chronic, non-communicable disease (NCD), and cavities resulting from untreated caries are among the most common unmet healthcare needs globally. Living without teeth affects quality of life and can lead to unhealthy diets, malnutrition and social isolation [2].

Dental caries occurs when plaque forms on the surface of a tooth and transforms the sugars contained in foods and drinks into acids that destroy the tooth over time [1]. It is formed when bacteria in the mouth metabolize sugars to produce acid that demineralizes the hard tissues of the teeth [3,4]. The magnitude of dental caries is still high [5], almost half of the world’s population, 3.5 billion people are affected, of which 40% remain untreated [3,6,7].

Dental caries is a global public health problem and is a widespread non-communicable disease that can be prevented. It affects a large number of people across all age and socioeconomic groups, affecting their health and well-being, social interactions and economic status [8–12]. It causes pain and difficulties in eating and sleeping, and result in chronic systemic infections. Dental caries are also associated with adverse growth patterns [13,14]. The treatment cost is expensive and is usually not part of the universal health coverage. The global economic cost of dental caries was estimated to be 442 billion USD per year, of which, 298 billion USD was spent on the treatment costs of dental caries and 144 billion USD was attributed to the lost working days [15].

Dental caries is expected to increase in many developing countries in Africa, particularly as a result of the growing consumption of sugars and inadequate exposure to fluorides [16]. There is a variation across countries and regions. According to WHO Regional Office for Africa, from thirty-nine sub-Saharan African countries, dental caries prevalence in children age twelve was 44%. In Ethiopia, the prevalence of dental caries ranges from 32.4% to 46.6% among the general population [17] and the prevalence of dental caries among adolescents is 32% [18]. Risks for the development of dental caries include environmental factors, nutritional factors, behavioral factors, socioeconomic and socio demographic factors. Other factors include oral salivary flow and composition, presence of cariogenic bacteria, immune components and genetic factors [19–22].

In Ethiopia, although the prevalence of dental caries at the national level is not clearly known, studies conducted in different parts of the country have shown an increase in prevalence among school children. This is because of factors associated with dental caries such as chewing khat, consumption of alcohol and cigarette smoking were highly practiced. Additionally, the choice of diversified nutrition was not practiced, and sweet foods such as biscuits, and sugar with coffee are widely used as contributing factors for dental caries [17,18].

Even though, everyone is at risk of dental caries, children and adolescents are at a higher risk of developing dental caries [5]. Different studies have reported a higher prevalence of dental caries in adolescents than in other age groups [23,24]. In addition, 25% of the Ethiopian population are adolescents [25]. Most permanent teeth erupted in these age groups. However, few studies have been conducted exclusively on adolescents concerning dental caries in Ethiopia. There is very little information on the factors associated with dental caries especially among adolescents. In light of these reports, this study aimed to identify factors associated with dental caries among adolescents in the west Wollega Zone public health hospitals.

## Methods

### Study setting and study period

This study was conducted in public hospitals in the West Wollega Zone, western Ethiopia. These Hospitals were Ayira Hospital, Gimbi Hospital, and Nedjo Hospital. The study was conducted from September 01, 2017 to October 01, 2017.

### Study design and population

A hospital-based unmatched case-control study was employed. The source population consisted of adolescent patients attending Hospitals in west Wollega, and the study population was adolescent patients attending the outpatient department of the selected hospitals. Cases were adolescents who visited the hospital for different services, and were diagnosed with dental caries. Controls were adolescents who visited the hospital and did not have dental caries on examination.

All adolescents who visited the hospital for different services, and fulfilled the definition of cases and controls were included in the study. Adolescents with mental illness and severe illness were excluded from the study. Furthermore, patients with dental caries outside of adolescent age group were excluded from the study. Also, adolescents with missed and filled teeth attributed to trauma were excluded from the study.

### Sample size and sampling procedure

The sample size was calculated using the double proportion formula on Epi Info version 7 based on the following assumptions; 95% two-sided Confidence level, 80% power and Ratio of case to control was 1:2 and from previous information, the proportion of tooth brushing habits among controls was 56% and odds ratio was 1.88 [26]. The calculated sample size was 399 (133 cases and 266 controls).

There were six public health hospitals in the West Wollega Zone. Three (50%) hospitals (Gimbi, Nedjo and Ayira Hospital) were selected using the lottery method. Then to select samples from each hospital, samples were proportionally allocated to each hospital, which was estimated based on their average patient flow for outpatient services in the month prior to data collection. Accordingly, one month prior to data collection, 856, 672 and 430 adolescent patients visited the OPD clinics of Gimbi, Nedjo, and Ayira general hospitals, respectively. After the sample was proportionally allocated to each hospital, systematic random sampling technique was used to select samples from each hospital with K = 5. K were calculated separately for the three health facilities. Finally, the first participants were selected using the lottery method for each hospital and for each case two consecutive controls were selected.

### Data collection techniques and data quality assurance

A structured questionnaire was developed to assess socio-demographic, lifestyle characteristics, nutritional factors, oral hygiene and knowledge of dental caries and its prevention among cases and controls of dental caries in adolescent patients attending selected hospitals. Questionnaires were adopted from different literature reviews [19,20,27,28]. An adopted questionnaire was translated into Afan Oromo and back translated to English by translators to ensure consistency. The questionnaire was pre-tested on 10% of the total sample size of the study at the Mendi Hospital. Some modifications concerning the clarification of the content and simplification of the wording were considered after the pre-testing of the questionnaire. The vague terms, phrases and questions identified during the pretest were modified and changed. The collected data were checked for completeness, accuracy, clarity, and consistency on a daily basis by a supervisor at each facility. Any errors and incompleteness were corrected before the data analysis.

Two data collectors (BSc Nurses in professional) and one supervisor (General practitioner) were selected from each hospital. Prior to data collection, data collectors and supervisors were trained on dental caries examination and assessment by dental health professionals and principal investigators in each hospital for one day. Each data collector and supervisor examined at least five adolescents under the supervision of a dental health professional to check their capacity to examine. Dental examinations were performed on all adolescents who visited the triage department and dental clinic of the facility to identify cases and controls.

Data were collected through face-to-face interviews. The collected data were checked daily by a supervisor in each hospital for completeness, clarity and consistency.

### Variable and outcome measurement

Dental caries was the dependent variable. It was assessed using the World Health Organization (WHO) dental caries diagnosis guideline by using the Decayed, Missing, and Filled Teeth (DMFT) index [28]. Dental examination was performed using a disposable glove, daylight, dental mirror, and wooden spatula. Clinical examinations for dental caries were performed by a dentist. A tooth was recorded as decayed when a carious lesion or both carious lesions and a restoration were present at the enamel or detectably softened wall or floor. The independent variables were socio-demographic factors, lifestyle characteristics of the patients, nutrition-related factors, and oral hygiene.

### Data management and analysis

Data were entered and cleaned using Epi Info software version 7, and then exported to the statistical package for social science (SPSS) software version 20 for analysis. Frequency and percentage were computed from univariable analysis to obtain summary values and assess the distribution. Bivariable analysis between the dependent and independent variables was performed separately using Bivariable binary logistic regression. In addition, a Chi-Square analysis was performed for each variable to assess whether significant differences were found between the case and control groups. Variables with p-value < 0.25 on bivariable analysis were candidate variables for multivariable analysis [29]. The strength of association between the dependent variable and independent variables was expressed as odds ratio (OR) with a 95% confidence interval. Finally, multivariable binary logistic regression analysis was performed to identify the independent effect of each independent variable on dental caries by accounting for the effect of others. The Multicollinearity assessment was conducted using the means of variance inflation factors (VIFs) as a post-estimation procedure following regression analysis. All covariates with a VIF <10 were tolerable and the presence of collinearity was declared if VIF >10 [30]. The Hosmer-Lemshow goodness of fit test was carried out and P-value > 0.05 ensured that the model adequately fitted the data and ROC curve was plotted for the fitted model and ROC with area under the curve of 0.70 and above were considered acceptable discrimination [29]. Finally, variables with P-values less than 0.05 on multivariable binary logistic regressions were considered as statistically significant associations with outcome variable.

### Ethical considerations

Ethical clearance was obtained from Wollega University and letters of co-operation were written to all selected hospitals and permission was secured at all levels. Written informed consent was obtained from each respondent aged 18 years and above and also written informed consent was obtained from their parents or guardians where participants are under 18 years of age.

## Results

### Socio-demographic characteristics of study participants

A total of 399 (133 cases and 266 controls) were interviewed and yielded a response rate of 100%. The number of females was 43 (32.3%) and 100 (37.6%) in cases and controls groups, respectively. Regarding educational status, 69(52%) of cases and 136(51.1%) of controls had attended high school or above educational level. Among study participants, 42/133(31.6%) of cases and 95/266(35.7%) of controls had an average monthly income < 500 ETB (about 18 USD). Ninety (67.7%) of cases and 126 (47.4%) controls were living in urban areas (Table 1).

**Table 1:** Socio-demographic characteristics of adolescents who attended selected hospitals in West Wollega, Western Ethiopia.

Variables	Category	Cases (%)	Controls (%)	Total (%)	χ^2^	P-value
Age (Years)	10–14	48(36.1)	107(40.2)	155(38.8)	0.638	0.424
	15–19	85(63.9)	159(59.8)	244(61.2)		
Sex	Male	90(67.7)	166(62.4)	256(64.2)	1.068	0.301
	Female	43(32.3)	100(37.6)	143 (35.8)		
Participant’s education	Elementary	64(48.1)	130(48.9)	194(48.6)	0.020	0.887
	Highschool or above	69(51.9)	136(51.1)	205(51.4)		
Paternal education	Illiterate	49(36.8)	96(36.1)	155(38.8)	0.216	0.898
	Elementary school	42(31.6)	90(33.8)	143(35.8)		
	High school or above	42(31.6)	80(30.1)	101(25.3)		
Maternal education	Illiterate	47(35.3)	108(40.6)	155(38.8)	1.547	0.461
	Elementary school	53(39.8)	90(33.8)	143(35.8)		
	High school or above	33(24.8)	68(25.6)	101(25.3)		
Ethnicity	Oromo	124(93.2)	249(93.6)	372(93.2)	0.386	0.825
	Amhara	6(4.5)	14(5.3)	20(5.0)		
	Others	3(2.3)	3(1.1)	7(1.8)		
Religious	Christian	102(76.7)	245(92.1)	347(87.0)	19.713	0.000
	Muslim	27(20.3)	16(6.0)	43(10.8)		
	Others	4(3.0)	5(1.9)	9(2.2)		
Marital status	Single	109(82.0)	221(83)	330(82.7)	0.079	0.779
	Married	24(18.0)	45(17)	69(17.3)		
Income	<500 ETB	42(31.6)	95(35.7)	137(34.3)	0.887	0.642
	500–1000 ETB	50(37.6)	99(37.2)	149(37.3)		
	>1000 ETB	41(30.8)	72(27.1)	113(28.3)		
Residence	Urban	90(67.7)	126(47.4)	216(54.1)	14.717	0.000
	Rural	43(32.3)	140(52.6)	183(45.9)		

### Life style characteristics, food habit, oral hygiene practice

Among study participants, 52(39.1%) of cases and 32(13.5%) of controls were reported to have chewed chat in their life. Regarding the history of alcohol intake, 23(17.6%) of cases and 32 (12.0%) of controls were reported they had a history of alcohol intake prior to the survey. The proportion of daily consumers of bread were higher in the cases 98 (73.7%) than controls 105(39.5%). Sixty-two (46.6%) of cases and 41(15.4%) of controls had drunk daily sugared coffee. In terms of daily toothbrush, 94 (70.7%) cases and 127 (47.0%) of controls did not brush their teeth on a daily base (Table 2).

**Table 2:** Life style characteristics, food habits and oral hygiene practice of adolescents in West Wollega, Western Ethiopia.

Variables	Category	Cases (%)	Controls (%)	Total (%)	χ^2^	P-value
Ever chewed Chat	Yes	52(39.1)	36(13.5)	88(22.1)	37.707	0.000
	No	81(60.9)	230(86.5)	311(77.9)		
Ever smoked	Yes	8(6.0)	5(1.9)	13(3.3)	4.811	0.028
	No	125(94.0)	261(98.1)	386(96.7)		
Ever drunk alcohol	Yes	23(17.3)	32(12.0)	55(13.8)	2.067	0.151
	No	110(82.7)	234(88.0)	344(86.2)		
Daily consumed bread	Yes	98(73.7)	105(39.5)	203(50.9)	41.522	0.000
	No	35(26.3)	161(60.5)	196(49.1)		
Use of injera as staple food	Yes	107(80)	205(77)	312(78)	0.595	0.440
	No	26(20)	61(23)	87(22)		
Daily drinking of tea	Yes	76(57.1)	130(48.9)	206(51.6)	2.429	0.119
	No	57(42.9)	136(51.1)	193(48.4)		
Daily drinking of sugared Coffee	Yes	62(46.6)	41(15.4)	103(25.8)	43.207	0.000
	No	71(53.4)	225(84.6)	296(74.2)		
Daily drinking of soft drink	Yes	33(24.8)	59(22.2)	92(23.1)	0.346	0.556
	No	100(75.2)	207(77.8)	307(76.9)		
Daily sweet food intake	Yes	77(57.9)	95(35.7)	172(43.1)	17.787	0.000
	No	56(42.1)	171(64.3)	227(56.9)		
Daily brush teeth	Yes	39(29.3)	139(52.3)	178(44.6)	18.871	0.000
	No	94(70.7)	127(47.7)	221(55.4)		
Mouth rinsing after any food	Yes	56(42.1)	108(40.6)	164(41.1)	0.083	0.774
	No	77(57.9)	158(59.4)	235(58.9)		

### Determinants of dental caries

On bivariable analysis, eleven variables were selected as candidate variables for multivariable analysis (P-value < 0.25) using the Chi-square test. Residence, daily consumption of sugared coffee, ever chewed chat, ever smoked habit, daily consumption of bread, and daily brushing of teeth were the factors found to be significantly associated with dental caries. There were no significant differences found in age group, sex, religion, marital status, income, and daily use of soft drinks between the case and control groups (Table 3).

**Table 3:** Determinants of dental caries among adolescents in west Wollega, Oromia regional state, Western Ethiopia.

Variables	Category	COR, 95%CI	AOR, 95%CI
Religion	Christian	0.24 (0.12,0.47)	0.56 (0.24,1.34)
	Muslim	0.52 (0.13,1.97)	0.25 (0.05,1.20)
	Others	Ref	Ref
Residence	Urban	2.32 (1.50,3.59)	1.86 (1.09,3.15) **
	Rural	Ref	Ref
Ever chewed Chat	Yes	4.10 (2.50,6.72)	2.90 (1.46,5.75) **
	No	Ref	Ref
Ever smoked	Yes	3.34 (1.07,10.42)	1.15 (0.26,5.10)
	No	Ref	Ref
Ever drunk alcohol	Yes	1.52 (0.85,2.73)	0.66 (0.29,1.49)
	No	Ref	Ref
Daily consumed bread	Yes	4.29 (2.71,6.78)	2.65 (1.44,4.89) **
	No	Ref	Ref
Daily drinking of tea	Yes	1.39 (0.91,2.12)	1.12 (0.66,1.90)
	No	Ref	Ref
Daily drinking of sugared Coffee	Yes	2.99 (1.84,4.88)	2.91 (1.62,5.23) **
	No	Ref	Ref
Daily Sweet food intake	Yes	2.47 (1.61, 3.78)	2.04 (1.19,3.48) **
	No	Ref	Ref
Daily brush teeth	Yes	0.37 (0.24,0.59)	0.48(0.28,0.81) **
	No	Ref	Ref

** Significance at 0.05 AOR-Adjusted Odds Ratio COR- Crude Odds Ratio.

Adolescents who daily drunk coffee with sugar were about 3 times (AOR = 2.91; 95%CI: 1.62, 5.23) more likely to develop dental caries than those who do not. An adolescent who reported to have chewed Chat was about 3 times (AOR = 2.90; 95%CI: 1.46, 5.75) more likely to develop dental caries than non-chewers. There was a significant association between daily consumption of bread and dental caries (AOR = 2.65; 95%CI: 1.44, 4.89). Also, daily consumption of sweet food was associated with dental caries (AOR = 2.04; 95%CI: 1.19, 3.48). Adolescents living in urban areas were 1.86 times more likely to have dental caries than those who live in a rural area (AOR = 1.86; 95% CI: 1.09, 3.15).

Adolescents who brush their teeth daily using tooth paste or chewable stick were less likely to have dental caries than those who did not (AOR = 0.48; 95% CI: 0.28, 0.81).

## Discussion

This study aimed to assess the determinants of dental caries among adolescents in public health facilities of West Wollega Zone. In this study, being living in an urban area, daily consumption of sugared coffee, ever chewed chat, ever smoked habit, daily consumption of bread, and lack of daily brush of teeth were the determinant factors of dental caries.

Adolescents who live in an urban area were more likely to have dental caries, in which odds of dental caries were higher. This finding is consistent with studies conducted in Ethiopia [31] and Uganda [32], in which living in an urban area is significantly associated with dental caries. This might be due to the higher consumption of sweet and processed foods for the urban adolescents than rural adolescents.

This study showed a strong association between dental caries and daily tooth brushing practices. This finding is supported by studies conducted in Addis Ababa, Gondor, and Finoteselam town of Ethiopia [26,33,34] in which teeth brushing prevented dental caries. In addition, a study conducted in Kenya on dental caries and its relation to oral care practice also reported less brushing of teeth increase dental caries [35]. This indicates brushing of teeth removes the food debris from the mouth and micro-organisms like Streptococcus mutants. This is due to the fact that the bacteria is unable to get enough nutrients and time for growth, thus, no acid production that causes dental caries development [15,36].

Adolescents who daily consumed sweet foods were more likely to develop dental caries than those who did not consume them. This finding was similar to a study conducted in Nairobi Kenya, Addis Ababa, and Finoteselam town [26,33,35]. Sugar plays a key role in increasing the prevalence of dental caries [37,38]. Sugar destructs teeth through the increased attachments sugar contents the teeth and finally making a conducive environment for bacterial growth. The bacteria then convert glucose, fructose, and most commonly sucrose (table sugar) into acids. If left in contact with the tooth, these acids cause demineralization of the hard tissues of the teeth which cause decay [39].

This study showed that, daily consumption of bread was one of the important predictors of dental caries. This might be due to higher contents of fermentable carbohydrates in bread which is the best media for bacteria to grow and saliva in the mouth breaks down the starch into sugar, then transformed into a gummy paste-like substance, the breadsticks to the crevices between teeth that can cause cavities [15,40]. This study indicated an adolescent who usually drinks coffees with sugar were more likely to develop dental caries than those who did not. This might be because of the higher content of carbohydrates in sugar, which may be left on teeth and become media for bacterial growth [15,41].

In this study, khat chewing was associated with dental caries. This was similar to a study conducted at Hawassa University, Ethiopia [21]. Khat is a leafy narcotic substance that is consumed by chewing the fresh leaves and consumption can lead to adverse oral effects including oral mucosal lesions, dryness of the mouth, discoloration of teeth, poor oral hygiene, and periodontal disease which in turn may predispose to dental caries [3]. Some studies showed that, chat chewers take much sugar, tea or coffee, or other sweet food when they chew which may enhance the association, however, it needs further studies and explanation [42–44].

This study has its own limitations. The selection of both cases and control were from hospitals that may ignore adolescents who did not attend the hospitals. Failure to use radiologic examination because of resource scarcities and using visual observation or clinical diagnosis to identify cases and controls was another limitation because visually undetected dental caries might be missed. Some assessments like food habits and lifestyles were based on the frequency of intake but the amount and duration were not well assessed. It was difficult to measure family income, mainly because many people are not comfortable disclosing income. Hence, for this study average monthly income was considered to assess economic status. In addition, only adolescent populations are included in this study which could not be generalizable to the general population.

## Conclusions and recommendations

This study found that living in urban area, Khat chewing habit, daily consumption of bread, daily consumption of sweet foods, and drinking of sugared coffee were risk factors for dental caries. However, daily brushing of teeth was found to protect adolescents from dental caries. We recommended that oral health programs should be incorporated into health extension programs. Comprehensive oral health education and proper oral hygiene should be practiced, and promotion of dietary choice and behavioral factors should be considered. Moreover, further studies are needed including hospitals, health centers, and private clinics as well as school or community-based studies using different methods of dental caries diagnosis, assessment of knowledge and practices of adolescents and other community groups on dental caries or overall oral health and risk factors should be conducted.

## Supporting information

S1 File SPSS dataset. (SAV)

## Abbreviations

AOR: Adjusted Odds Ratio
CI: Confidence Interval
COR: Crude Odds Ratio
DMFT: Decayed, Missing and Filled permanent teeth
E.C: Ethiopian Calendar
ETB: Ethiopian Birr
Km: Kilometer
NGO: Non-Governmental Organization
OR: Odds Ratio
SPSS: Statistical package of social science
WHO: World Health Organization
